# Study of the Relationship Between Pelvic Organ Prolapse Quantification (POP-Q) Staging and Decubitus Ulcer in Pelvic Organ Prolapse

**DOI:** 10.7759/cureus.12443

**Published:** 2021-01-03

**Authors:** Subha R Samantray, Ipsita Mohapatra

**Affiliations:** 1 Obstetrics and Gynecology, Prathima Institute of Medical Sciences, Karimnagar, IND; 2 Obstetrics and Gynecology, Prathima Institute of Medical Sciences, Kaloji Narayana Rao (KNR) University of Health Sciences, Karimnagar, IND

**Keywords:** pelvic organ prolapse, decubitus ulcer, pop-q staging

## Abstract

Introduction

The protrusion of pelvic organs and their associated vaginal segments into or through the vagina is called pelvic organ prolapse (POP). One of the worrisome complications of POP is decubitus ulcer.It is a feature of old, long-standing prolapse. The standardized pelvic organ prolapse quantification (POP-Q) system of classification was proposed by the International Incontinence Society (ICS) for staging pelvic organ prolapse in 1996. This system is a clear and reproducible quantification method and has more inter-observer and intra-observer reliability. This study was designed to determine the relationship of the POP-Q stage with the presence and size of decubitus ulcer.

Methods

A total of 92 cases with pelvic organ prolapse were included in the study. We examined our patients by the POP-Q method and measured the dimension of the decubitus ulcer if present.

Results

Out of the 92 patients included in the study, 32 cases had a decubitus ulcer. As the POP-Q stage increased, the number of patients with a decubitus ulcer also increased. We also observed that larger dimension (>6 cm^2^) decubitus ulcers are present in stage 3 and stage 4 POP. A statistically significant correlation was observed between the POP-Q stage and the size of the decubitus ulcer, r_s_=0.607, p<0.001.

Conclusion

A decubitus ulcer becomes more common as the POP-Q stage increases. The size of the ulcer also increases with the advancing stage of POP-Q.

## Introduction

The protrusion of pelvic organs and their associated vaginal segments into or through the vagina is called pelvic organ prolapse (POP). It is a common, distressing, and incapacitating condition that affects about 30% of women worldwide [1]. POP results due to tears in the supports of the uterus and vagina, neuromuscular dysfunction, or both. Damage to the supporting structures may result due to difficult and prolonged labor, premature bearing down by the mother, fundal pressure, or inappropriate traction forces by trained or untrained birth attendants. Sometimes, it may also occur due to congenital weakness of the connective tissue supporting structures [2]. There is an increase in prolapse rate with an increase in age, parity, and menopause [3-4].

Prolapse is generally associated with the increasing age of the woman due to hypoestrogenic states. It is also more common in women of lower economic status due to inaccessibility to health care facilities, home deliveries that are not attended by skilled persons, multiple and frequent pregnancies, and poor nutritional status of the mothers.

One of the frequent complications of POP is decubitus ulcer [5-6]. Its incidence ranges from 3% to 50% [7-8]. It is a feature of old, long-standing prolapse. The frequent inciting factors are venous congestion and localized trauma [6]. There is edema and an increase in the friability of the tissue. Sometimes, these ulcers may be associated with cervical intraepithelial neoplasia and cervical malignancy [9-10]. The presence of a decubitus ulcer makes surgical correction of prolapse difficult; hence, it needs to be treated before surgery. Estrogen-soaked vaginal packing is used to promote decubitus ulcer healing [5]. Glycerine and betadine packs are also useful in treating decubitus ulcers.

There are various classification systems for POP. But most of these systems lacked standardization and there was a lot of inter-observer difference. In 1996, the pelvic organ prolapse quantification (POP-Q) system of classification was proposed by the International Incontinence Society (ICS) [11]. This system is a clear and reproducible quantification method and has more inter-observer and intra-observer reliability. According to this system, nine points are taken. There are two anterior points (Aa and Ba), two posterior points (Ap and Bp), and two apical points (C and D). The other three measurements are total vaginal length (tvl), genital hiatus (gh), and perineal body (pb). All these measurements are taken in centimeters with the hymen as the reference plane. Any point proximal to the hymen is considered as negative and distal to the hymen as positive. The hymen level is referred to as 0.

The prolapse is staged according to the point that is most distal.

Stage 0 - no prolapse, anterior and posterior points are all -3 cm, C and D are between -tvl and - (tvl-2) cm

Stage 1 - the distal prolapse is more than 1 cm above the level of the hymen (less than -1 cm)

Stage 2 - the most distal prolapse is between 1 cm above and 1 cm below the hymen

Stage 3 - the most distal prolapse is more than 1 cm below the hymen and no further than (tvl-2) cm

Stage 4 - complete procidentia or vault eversion, the most distal prolapse is at least (tvl-2) cm

In this study, we tried to find the relationship between the POP-Q stage and the presence of decubitus ulcer. An attempt was also made to find out the association, if present, between the POP-Q stage and the size of the ulcer.

## Materials and methods

This is a prospective observational study carried out at Prathima Institute of Medical Sciences in southern India from June 2018 to May 2020. The study was approved by the institutional ethical committee. During this study period, a total of 106 cases came to the gynecology outpatient department with the symptoms of pelvic organ prolapse. All patients who had symptoms and signs of pelvic organ prolapse were enrolled. Those who gave consent for the study were included in the study. Vault prolapse and patients having any cervical pathology were excluded from the study. Patients with any other kind of prolapse like rectal prolapse were also excluded.

**Figure 1:**
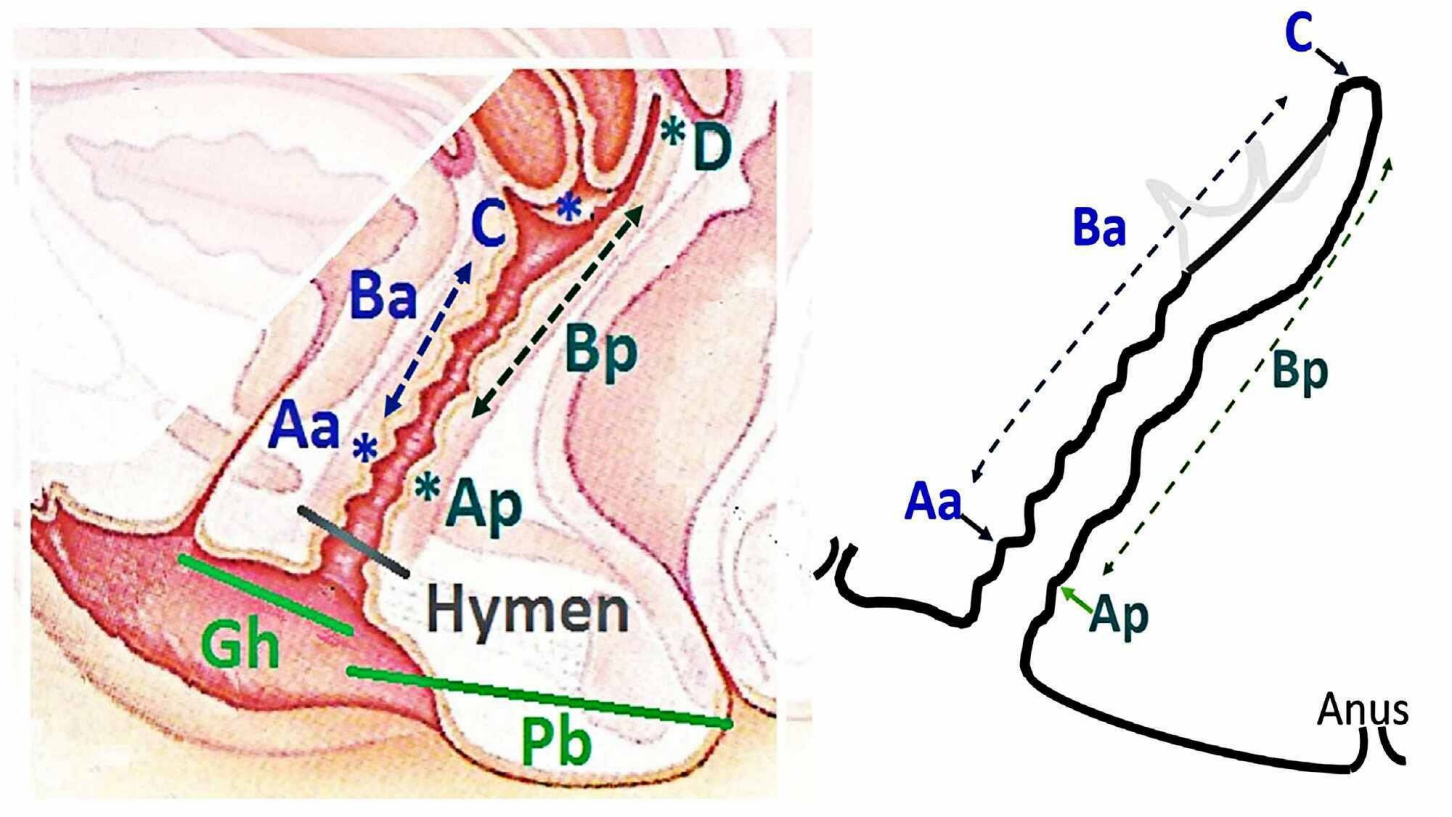
POP-Q system of classification* Nine points are used in the POP system of quantification. Aa and Ap are 3 cm proximal to the hymen when POP is fully reduced. Ba and Bp reflect the lowest point reached by a POP in the complete prolapsed state. C is the most distal edge of the cervix or vaginal cuff (post-hysterectomy). D is the posterior fornix (if the uterus is in situ). Gh is genital hiatus, Pb is perineal body, tvl is total vaginal length (not shown in the figure). *Adapted from Madhu C et al. How to use the pelvic organ prolapse quantification (POP-Q) system? Neurourology and Urodynamics. 2018;37:S39–S43, with permission from "John Wiley and Sons". [12]

Informed consent was taken from all participants. Data regarding social demographic characteristics, reproductive history, and pelvic examination findings were obtained in a predesigned proforma. Patients were examined in the dorsal lithotomy position and with an empty bladder. The prolapse was staged according to the POP-Q method, as illustrated in Figure 1 and all the measurements were taken in centimeters with reference to the hymen [12].

If a decubitus ulcer was present, its number and dimension were measured. For multiple ulcers, the sum of all areas was taken. The patients were divided into small size (0 - 3 cm²), medium size (>3-6cm²), and large size (>6cm²) for the study. Data of all patients were collected, tabulated in a Microsoft Excel spreadsheet. Data analysis was carried out using the Statistical Package for the Social Sciences (SPSS) version 21 (IBM Corp., Armonk, NY). Categorical variables were described as frequency and proportion. Proportions were compared using the chi-square and Fisher exact test, whichever was applicable. All quantitative variables were estimated using measures of central location and measures of dispersion. For normally distributed data, the mean was compared using the student’s t-test and for skewed data, the Mann-Whitney U test was used for two groups. Spearman’s correlation coefficient was used to assess the relationship between the stage of prolapse and the size of decubitus ulcers. For all statistical analyses, a p-value of <0.05 was considered significant.

## Results

Out of the 106 patients with prolapse, 13 cases had vault prolapse and one case was a post-radiotherapy case of carcinoma cervix. A total of 92 cases were included in this study.

Table 1 shows the demographic variables of patients with prolapse. The mean age of occurrence of prolapse in our study was 56.86±9.5 years. Prolapse was more common in women with higher parity (74% of cases were reported in women who had three or more deliveries). A lower socioeconomic status was more commonly associated (84%) with POP. Most of the cases (86%) had a rural background.

**Table 1:** Demographics of patients with pelvic organ prolapse POP-Q: pelvic organ prolapse quantification

Demographics of patients with pelvic organ prolapse		
Variables		Frequency (%)
Age in years		56.86 ± 9.5
Parity	1-2	24 (26.1%)
	3-4	48 (52.2%)
	≥5	20 (21.7%)
Socioeconomic status	Lower	77 (83.7%)
	Middle	14 (15.2%)
	Upper	1 (1.1%)
Residence	Rural	79 (85.9%)
	Urban	13(14.1%)
Place of delivery	Home	51 (55.4%)
	Hospital	29 (31.5%)
	Home & Hospital	12 (13.1%)

**Figure 2:**
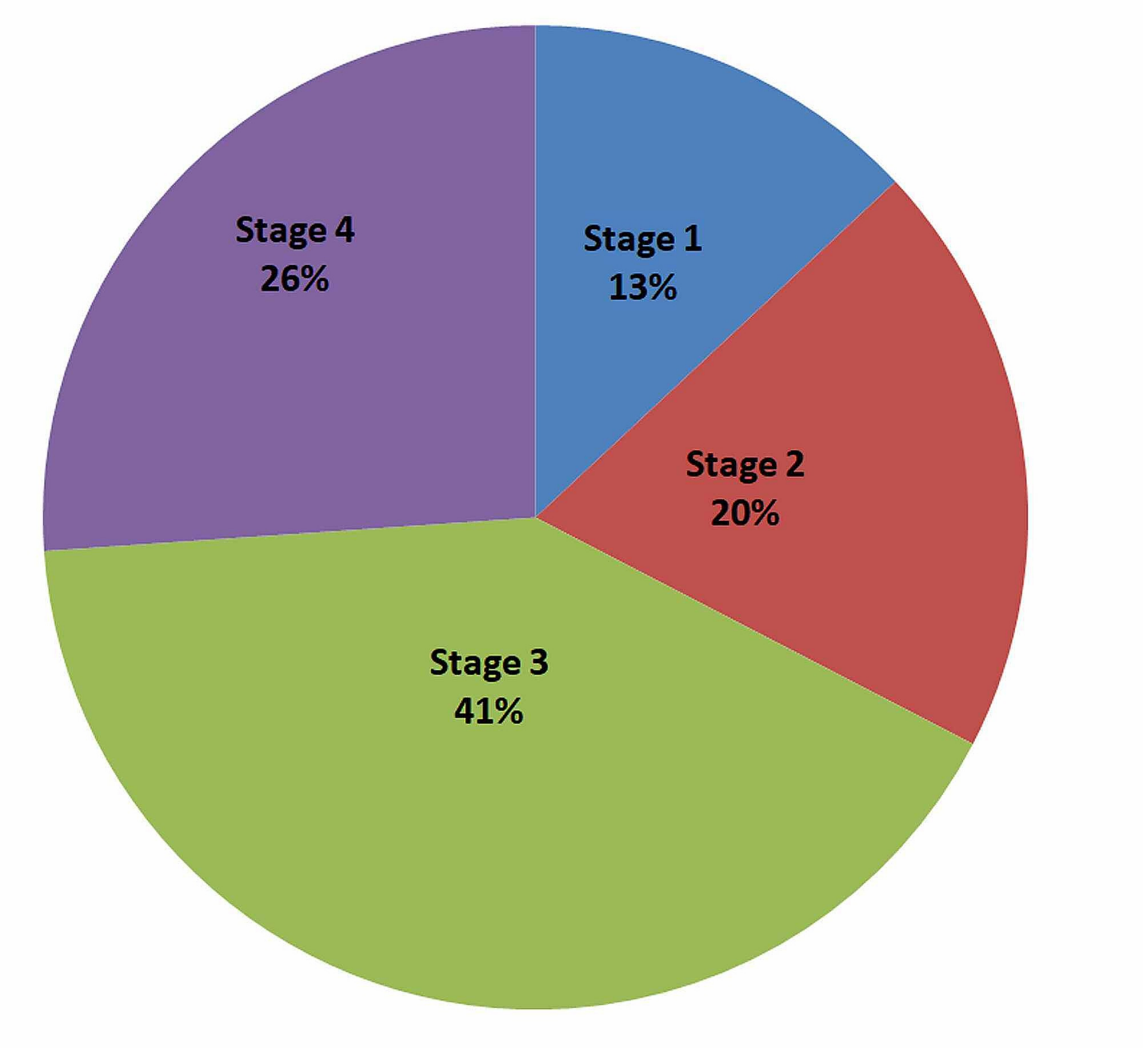
Distribution of cases according to POP-Q staging POP-Q: pelvic organ prolapse quantification

Figure 2 depicts the distribution of all the cases according to their POP-Q staging. The maximum number of cases had POP-Q stage 3 (41%) followed by POP-Q stage 4 (26%).

In Table 2, the number of prolapse patients with decubitus ulcers is shown. A total of 32 cases had decubitus ulcers. Of these, stage 4 had the maximum number of cases with decubitus ulcer (18 cases) while none of the stage 1 patients had decubitus ulcer.

**Table 2:** Distribution of cases of decubitus ulcer according to POP-Q stage POP-Q: pelvic organ prolapse quantification

Distribution of cases of decubitus ulcer according to POP-Q stage			
POP-Q stage	Number of cases with decubitus ulcer (n =32)	Number of cases without decubitus ulcer(n =60)	Total
Stage 1	0	12	12
Stage 2	2	16	18
Stage 3	12	26	38
Stage 4	18	6	24
Total	32	60	92

Table 3 shows the relationship of the POP-Q stage with the dimension of the decubitus ulcer. Greater than 6 cm^2^ ulcers are seen maximum in stage 4 (50%), whereas no decubitus ulcer is observed in stage 1. 6.3% of cases of stage 2 had ulcers with a size of less than 3 cm^2^. Spearman’s rho correlation coefficient (r_s_) was used to assess the relationship between the POP-Q stage and the size of the decubitus ulcer, a significant correlation was observed between them, r_s_ =0.607, p<0.001.

**Table 3:** Relationship of the POP-Q stage with the dimension of the decubitus ulcer *Stage 1 is not included, as no cases presented with decubitus ulcer

Relationship of POP-Q stage with the dimension of the decubitus ulcer				
Area of decubitus ulcer	Stage 2*	Stage 3	Stage 4	Total
< 3 cm^2^	2 (6.3%)	4 (12.5%)	0 (0%)	6 (18.8%)
3-6 cm^2^	0 (0%)	7 (21.9%)	2 (6.3%)	9 (28.1%)
>6 cm^2^	0 (0%)	1 (3.1%)	16 (50%)	17 (53.1%)
Total	2 (6.3%)	12 (37.5%)	18 (56.3%)	32 (100%)

Table 4 depicts the comparison of pelvic organ prolapse patients' characteristics between groups with and without decubitus ulcer. No statistically significant difference was observed with respect to age, socioeconomic status, place of delivery, parity, menopausal status, parity, etc. Comparison of body mass index (BMI) between the group with decubitus ulcer (M=28.2, SD= 3.98) and the group without decubitus ulcer (M=24.91, SD=3.84) demonstrates significantly higher BMI among patients with decubitus ulcer, t (90) =4.77, p<0.001.

**Table 4:** Characteristics of groups with and without decubitus ulcer * Statistically significant BMI: body mass index

Characteristics of groups with and without decubitus ulcer				
		Cases with decubitus ulcer (n=32)	Cases without decubitus ulcer (n=60)	p-value
Age (in years)		57.4±9.9	55.84±8.4	0.455
Socioeconomic status	Lower	24(75%)	53(88.3%)	
	Middle	8(25%)	6(10%)	
	Upper	0(0%)	1(1.7%)	
Residence	Rural	26(81.2%)	53(88.3%)	0.364
	Urban	6(18.8%)	7(11.7%)	
Place of delivery	Home	22(68.7%)	29(48.3%)	
	Hospital	8(25%)	21(35%)	
	Home & hospital	2(6.3%)	10(16.7%)	
Menopausal status	Premenopausal	9(28.1%)	14(23.3%)	0.801
	Postmenopausal	23(71.9%)	46(76.7%)	
Years since menopause (in years)		10.9±6.3	7.9±5.6	0.056
BMI (kg/Mt^2^)		26.27±3.18	23.18±2.8	<0.001^*^

## Discussion

POP-Q represents the joint collaboration of several investigations and was adapted by the International Incontinence Society (ICS), the American Urogynecologic Society (AUGS), and the Society of Gynecologic Surgeons (SGS) and was finally accepted as the first internationally recognized POP classification system [13]. In the POP-Q technique, the measurements are taken in centimeters. The nine points are well-defined and so there is very less inter-observer and intra-observer variation. Because of its high accuracy, it is preferred for research purposes.

In our study, the mean age of women presenting with uterovaginal prolapse was 56.86±9.5 years. The mean age in our study is comparable to the study done by Isikhuemen ME et al., where the mean age was 56.37 years [14]. Prolapse is more common in older age women. The Pelvic Organ Support study found age to be a risk factor for pelvic organ prolapse and risk doubled with each decade of life [15]. The rate of prolapse also tends to increase with the increase in parity. The Oxford Family Planning study analyzed 17,000 women and concluded that those with a history of two vaginal deliveries were 8.4 times more likely to have surgery for prolapse than those with no such history [16]. According to an observation by Quiroz LH et al., the odds of pelvic organ prolapse were almost 10 times higher after a single vaginal birth with a marginal impact of additional births on this association [17]. Damage to supporting structures, neuromuscular dysfunction, or both occurring during difficult or non-institutional deliveries are known causes of pelvic organ prolapse. In our study, 70% of the patients were having parity of order three or more, ranging from one to eight. Most of the patients were from lower socioeconomic status (84%). This might be due to the lack of access to a healthcare system during pregnancy and childbirth, inadequate rest in the postpartum period, early resumption of physical activity after childbirth, and lack of proper nutrition in women of low socioeconomic strata. Similarly, prolapse was more common in women from rural backgrounds (86%). Lifestyle and occupational activities in rural areas of low-income countries, which include lifting heavy weights, direct the pressure on pelvic floor muscles to cause prolapse.

We also observed that prolapse was more common in women who had a home delivery than women who had their delivery at hospitals. This might be because of unattended births at home or the use of improper techniques used by untrained midwives or dais attending the home deliveries. Lack of proper care during deliveries is one of the major risk factors for prolapse.

In our study, we observed that there is an increase in the incidence and size of decubitus ulcer as the POP-Q stage increases. Most of the cases neglected themselves and presented late with a higher stage of pelvic organ prolapse and a larger size of decubitus ulcer. A similar result has also been shown by Deshpande et al. [18]. Deshpande et al. also observed that as the measurement of point C increases, the area of decubitus ulcer also increases.

The only problem with the POP-Q technique is its complexity for the beginners to understand and the time consumed for the total examination. A six-point simplified version of POP-Q was suggested by Raizada N et al. as SPOP-Q [19].

When we compared prolapse with decubitus ulcer and various other factors, we observed that decubitus ulcers are more common in old patients. Old patients and postmenopausal patients have low estrogen levels, which can be the reason behind mucosal atrophy, venous congestion, and decubitus ulcer. Similar results were reported by Isikhuemen ME et al. [14]. A decubitus ulcer is more seen in patients coming from low socioeconomic status. The reason behind this may be poor nutrition, lack of proper hygiene, and seeking medical advice only after the symptoms become severe, that is, when the prolapse is either stage 3 or 4. When compared for various risk factors, no difference was observed among the groups with and without decubitus ulcer except body mass index. A significantly higher BMI was observed in patients with decubitus ulcers. Systematic review and meta-analysis by Giri A et al. demonstrated that overweight and obese women are more likely to have pelvic organ prolapse as compared with women with body mass index in the normal range [20].

## Conclusions

A decubitus ulcer is a very common association of pelvic organ prolapse, but it has not been studied as extensively. Decubitus ulcers become more common as the POP-Q stage increases. The size of the ulcer also increases with the advancing stage of POP-Q. So if we find a decubitus ulcer during the examination of a prolapse patient, we have to correct it first. It should be treated properly before a surgical procedure is done for better postoperative results and fewer difficulties during the operative correction of pelvic organ prolapse.
